# A Novel Missense Mutation of the NSD1 Gene Associated with Overgrowth in Three Generations of an Italian Family: Case Report, Differential Diagnosis, and Review of Mutations of NSD1 Gene in Familial Sotos Syndrome

**DOI:** 10.3389/fped.2017.00236

**Published:** 2017-11-07

**Authors:** Gianluigi Laccetta, Francesca Moscuzza, Angela Michelucci, Andrea Guzzetta, Sara Lunardi, Francesca Lorenzoni, Paolo Ghirri

**Affiliations:** ^1^Department of Maternal and Child Health, Division of Neonatology and Neonatal Intensive Care Unit, Santa Chiara Hospital, University of Pisa, Pisa, Italy; ^2^Division of Cytogenetic and Molecular Biology, Azienda Ospedaliera Universitaria Pisana (AOUP), Pisa, Italy; ^3^Department of Developmental Neuroscience, IRCCS Stella Maris, University of Pisa, Pisa, Italy

**Keywords:** Sotos, NSD1, overgrowth, dolichocephaly, learning disability, cryptorchidism, hypertelorism

## Abstract

Sotos syndrome (SoS) is characterized by overgrowth of prenatal onset, learning disability, and characteristic facial appearance; it is usually due to haploinsufficiency of NSD1 gene at chromosome 5q35. An Italian child was born at 37 weeks of gestation (weight 2,910 g, 25th–50th centiles; length 50 cm, 75th centile; head circumference 36 cm, 97th centile) showing cryptorchidism on the right side, hypertelorism, dolichocephaly, broad and prominent forehead, and narrow jaw; the pregnancy was worsened by maternal preeclampsia and gestational diabetes, and his mother had a previous history of four early miscarriages. The patient showed neonatal jaundice, hypotonia, feeding difficulties, frequent vomiting, and gastroesophageal reflux. After the age of 6 months, his weight, length, and head circumference were above the 97th centile; psychomotor development was delayed. At the age of 9 years, the patient showed also joint laxity and scoliosis. DNA sequence analysis of NSD1 gene detected a novel heterozygous mutation (c.521T>A, p.Val174Asp) in exon 2. The same mutant allele was also found in the mother and in the maternal grandfather of the proband; both the mother and the maternal grandfather of the proband showed isolated overgrowth with height above the 97th centile in absence of other features of SoS. At present 23 familial cases of SoS have been described (two cases with mutation in exon 2 of NSD1 gene); no familial cases of SoS with mutation of NSD1 gene and isolated overgrowth have been reported. Probably, point mutations of NSD1 gene, and particularly mutations between exon 20 and exon 23, are not likely to affect reproductive fitness. Epigenetic mechanisms and intrauterine environment may influence phenotypes, therefore genetic tests are not useful to predict the phenotype but they are indispensable for the diagnosis of SoS. This is the first Italian familial case of SoS with genetic confirmation and the third report in which a missense mutation of NSD1 gene is found in three generations of the same family.

## Background

Sotos syndrome (SoS) was first described by Juan Sotos in 1964; it is a rare genetic disorder whose prevalence is estimated to be 1:14,000 live births (1, 2).

Sotos syndrome is characterized by overgrowth of prenatal onset, learning disability, and characteristic facial appearance: it has been estimated that at least 90% of patients with SoS present these features (2). The head of these patients is typically dolichocephalic, and the forehead is broad and prominent; palpebral fissures are downslanting, the jaw is narrow with a long chin and hair in the frontotemporal region is sparse (2). Children with SoS usually present delay of motor development, hypotonia, and poor language; they also have difficulties with speech and language, and intellectual impairment that remains stable throughout life (2). The average birth length approximates to the 98th centile, the average birth weight is between the 50th and 91st centiles, and the average birth head circumference is between the 91st and 98th centiles (2). Even though growth is influenced by parental heights, children with SoS usually have a height and head circumference at least two SD above the mean (2). Height can be normal in adulthood, but macrocephaly is usually present at all ages (2). The increased height is usually due to an increase in limb length; 75–80% of children also present with advanced bone age (2).

Less frequently, patients with SoS present with behavioral problems such as autistic spectrum disorders, phobias, and aggression (2). Cardiac anomalies are identified in 20% of patients (2). Most of individuals with SoS have cranial abnormalities found on MRI or CT (ventricular dilatation, hypoplasia or agenesis of the corpus callosum, mega cisterna magna, cavum septum pellucidum, cerebral atrophy, and small cerebellar vermis) (2). Joint laxity has been described in at least 20% of patients with SoS; affected individuals often present renal anomalies, scoliosis, and seizures (2). 15% of pregnancies of children with SoS are worsened by maternal preeclampsia, and neonatal complications (jaundice, hypotonia, and poor feeding) are very common (2).

Less than 15% of individuals with SoS present with cataract, conductive hearing loss, craniosynostosis, vertebral anomalies, talipes equinovarus, hemihypertrophy, gastroesophageal reflux, hypothyroidism, and cryptorchidism (2). Tumors (sacrococcygeal teratoma, neuroblastoma, presacral ganglioma, acute lymphoblastic leukemia, small cell lung cancer, and soft-tissue sarcoma) have been described in about 3% of patients with SoS (2, 3).

In 2002, cloning of the breakpoints of a “*de novo*” t(5;8)(q35;q24.1) translocation in a child with SoS led to the discovery that SoS is usually due to haploinsufficiency of the NSD1 gene that encodes a histone methyltransferase (the nuclear receptor SET domain-containing protein 1); the NSD1 gene is located at chromosome 5q35, and its haploinsufficiency is caused by various mechanisms (truncating mutations, missense mutations, splice-site mutations, partial gene deletions, and 5q35 microdeletions) (1, 2). The histone–lysine *N*-methyltransferase encoded by the NSD1 gene acts as a transcriptional factor capable of both negatively and positively influencing transcription, depending on the cellular context (2). Intragenic mutations cause 80–85% of SoS cases among European and American populations; 5q35 microdeletions are present in 10–15% of European and American cases and in more than 50% of Japanese cases (1). No abnormalities of the NSD1 gene have been identified in 10% of patients (1). Many benign and pathogenic variants of *NSD1* have been identified, but it is not currently known how functional abrogation of *NSD1* results in SoS; probably there is a link between SoS and MAPK/ERK-signaling pathway, which is downregulated in SoS (2, 4). On the other side, the ras interacting protein 1, a downstream Ras effector interfering with the MAPK/ERK pathway, is identified upregulated in SoS (4). The deregulation of the MAPK/ERK-signaling cascade causes a hypertrophic differentiation of *NSD1*-expressing chondrocytes with subsequent statural overgrowth and accelerated skeletal maturation in patients with SoS (4).

Furthermore, heterozygous inactivation of the NSD1 gene results in loss of repression of growth promoting genes, modestly increased plasma levels of IGFBP-2 and IGFBP-6, and reduced levels of IGF-I, IGF-II, IGFBP-3, and IGFBP-4 (4). Reduced levels of IGF-I and IGF-II are present in short rather than tall stature such as observed in SoS, therefore the relationship between *NSD1* and the GH/IGF1 axis is still unclear (4).

In this report, we describe a novel molecular genetic finding in the NSD1 gene with related clinical features in an Italian familial case of overgrowth syndrome.

## Case Presentation

The patient was the second child of non-consanguineous healthy parents of Italian origin with a history of four early miscarriages. He was born at 37 weeks of gestation by spontaneous delivery; the pregnancy was affected by maternal preeclampsia and gestational diabetes. His birth weight was 2,910 g (between the 25th and 50th centiles), his length was 50 cm (75th centile), and his head circumference was 36 cm (97th centile). Apgar score was 8 at 5 min. Physical examination performed at birth revealed cryptorchidism on the right side, hypertelorism, dolichocephalic head with broad and prominent forehead, and narrow jaw. Blood glucose monitoring in the 24 h following the birth was normal; 48 h after birth the newborn showed neonatal jaundice that required phototherapy. Cardiac and abdominal ultrasound examinations were normal; cerebral ultrasound study showed a mild periventricular hyperechogenicity.

The patient showed hypotonia and severe feeding difficulties since he was 3 weeks old; at the age of 1 month, gastroesophageal reflux was diagnosed, and frequent vomiting was present since he was 6 weeks old. At the age of 7 weeks, the child had an episode of Brief Resolved Unexplained Event with cyanosis, absent breathing, marked hypotonia, and unresponsiveness. Later, he showed some episodes of apnea with perioral cyanosis. For these reasons, the patient underwent cardiorespiratory monitoring and artificial feeding with nasogastric tube for 10 days.

The weight gain of the patient was poor until the age of 6 months because of hypotonia, feeding difficulties, gastroesophageal reflux, and frequent vomiting. Later, he showed excessive growth, and his weight, height, and head circumference were above the 97th centile for age and sex. Psychomotor development was delayed: the child held up his head at 5 months, rolled over at 7 months, walked alone at 24 months, spoke a first word at 30 months and simple phrases at 4 years. At 4 years 6 months, he showed advanced bone age corresponding to 6 years.

At the age of 9 years, the patient weighed 51.4 kg (>97th centile); he was 148.1 cm tall (>97th centile), and his head circumference was 60 cm (>97th centile). Physical examination revealed dolichocephaly, broad and prominent forehead, downslanting palpebral fissures, narrow jaw, hypertelorism, increased limb length, joint laxity, and scoliosis; his right testicle was smaller than his left one. Intellectual impairment and difficulties with speech and language were noted. Sideropenic anemia was demonstrated at blood examination.

Clinical findings, after a differential diagnosis with other overgrowth syndromes (Table 1), supported the suspicion of SoS, thus molecular genetic analysis was recommended.

**Table 1:** Suggestive findings of Sotos syndrome (SoS) in the proband and differential diagnosis with other overgrowth syndromes (1 = Malan syndrome; 2 = Marshall-Smith syndrome; 3 = Weaver syndrome; 4 = Simpson–Golabi–Behmel syndrome; 5 = Perlman syndrome; 6 = Bannayan–Riley–Ruvalcaba syndrome; 7 = Beckwith–Wiedemann syndrome; 8 = PI3K-related syndromes; + = present; − = absent; NA = not available).

Suggestive findings for SoS	Proband	Other overgrowth syndromes							
		1	2	3	4	5	6	7	8
Broad, prominent forehead	+	+	+	+	+	+	−	−	+
Dolichocephalic head shape	+	−	−	−	−	−	−	−	−
Sparse frontotemporal hair	−	−	−	−	−	−	−	−	−
Downslanting palpebral fissures	+	+	−	−	+	−	−	−	−
Malar flushing	−	−	−	−	−	−	−	−	−
Long narrow face	+	+	−	−	−	−	−	−	−
Long chin	−	+	−	−	−	−	−	−	−
Early developmental delay	+	+	+	+	+	+	+	+	+
Mild/severe intellectual impairment	+	+	+	+	−	−	+	−	+
Height ≥2 SD above the mean	+	+	−	+	+	+	+	+	−
Head circumference ≥2 SD above the mean	+	+	−	+	+	−	+	+	+
Normal height in adulthood	NA	+	−	−	−	+	+	+	+
Macrocephaly at all ages	NA	+	−	+	+	−	+	−	+
Behavioral problems	−	+	−	−	+	−	+	−	+
Advanced bone age	+	+	+	+	−	+	−	−	−
Cardiac anomalies	−	−	−	+	+	+	−	−	−
Cranial MRI/CT abnormalities	−	−	−	+	+	+	−	−	+
Joint hyperlaxity/pes planus	+	−	−	−	−	−	−	−	−
Maternal preeclampsia	+	−	−	−	−	−	−	−	−
Neonatal complications	+	+	+	+	+	+	+	+	−
Renal anomalies	−	−	−	−	+	+	−	+	−
Scoliosis	+	+	−	−	+	−	−	−	+
Seizures	−	+	−	−	+	−	+	−	+

*See Ref. (2, 5–7)*.

## Laboratory Investigations and Diagnostic Tests

Genomic DNA was extracted from peripheral blood lymphocytes using EZ1 DNA kit and BioRobot EZ1 (Qiagen, Milan, Italy). All coding exons (exons 2–23) and exon–intron boundaries of the NSD1 gene (Ensembl Gene ID: ENSG00000165671, ENST00000439151) were amplified by polymerase chain reaction (PCR) (primers sequences and PCR conditions are available on request). PCR products were purified with QIAquick PCR purification kit (Qiagen, Valencia, CA, USA) and directly sequenced with Big Dye Terminator Sequencing Kit version 3.1 (Applied Biosystems, Foster City, CA, USA), with the same primers used for the amplification. Finally, samples were run on 3130xl Genetic Analyzer (Applied Biosystems, Foster City, CA, USA) and analyzed with Sequencing Analysis Software (Applied Biosystems, Foster City, CA, USA).

DNA sequence analysis of the NSD1 gene detected a novel heterozygous mutation: c.521T>A (p.Val174Asp) in exon 2. The same mutant allele was also found in the mother and in the maternal grandfather of the proband. *In silico* analysis by PolyPhen-2 predicted it as probably damaging; analysis with Mutation Taster predicted the mutation as disease causing. The residue is conserved among different species.

The mother was the only child of unrelated parents. She was 45 years old at diagnosis with height of 181.6 cm (>97th centile); her weight and head circumference were normal. She had neither facial appearance suggestive of SoS nor intellectual impairment; she graduated at University and worked as employee. She was born as appropriate-for-date, no neonatal complications were observed, and her psychomotor development was normal. She suffered from Hashimoto thyroiditis and had a previous history of four early miscarriages.

The maternal grandfather of the patient was 79 years old at diagnosis with height of 195.4 cm (>97th centile); his weight, head circumference, and facial appearance were normal. He was born as appropriate-for-date, no neonatal complications were observed, and his psychomotor development was normal. He graduated at senior high school with good results and had worked as employee.

At present, the child undergoes periodic clinical examination and investigations because of the increased risk of tumors and other complications; orthopedic follow-up for scoliosis is also performed. The child participates in a regular class with support, and his parents undergo periodic psychological counseling.

Written informed consent for the publication of the present case report was obtained from both parents of the proband and his maternal grandfather.

## Discussion

We hypothesized our patient should suffer from SoS because of his history of overgrowth, his facial appearance, and the presence of many other suggestive findings (Table 1) (2, 5–7). However, a differential diagnosis with other overgrowth syndromes was mandatory: a lot of the suggestive findings showed by our patient were also characteristic of many other overgrowth syndromes but, finally, the history of maternal preeclampsia and the presence of dolichocephaly and joint laxity let us suspect SoS (Table 1) (2, 5–7). However, the definitive diagnosis required necessarily the sequence analysis of NSD1 gene.

In our family, the same mutation in exon 2 of the NSD1 gene was present in the proband, in his mother and in his maternal grandfather; all the three patients showed overgrowth. In addition to overgrowth, the proband showed most of the features of SoS. At present 23 families with at least two family members affected by SoS have been identified as carrier of mutations of NSD1 gene associated with overgrowth and other clinical findings of SoS (Table 2) (8–18). Ho¨glund et al. reported a Finnish family with two members showing the classical phenotype of SoS: both of them had a novel 896delC mutation at exon 2 resulting in a frameshift and premature stop codon at nucleotides 955–957 with truncation of 88% of the predicted polypeptide (9). de Boer et al. reported a family with the eldest son showing advanced bone age, developmental delay, and some facial characteristics of SoS, and his mother showing overgrowth and some facial characteristics of SoS; both the mother and the son were carriers of the missense mutation 607G>A at exon 2 of NSD1 gene (11).

**Table 2:** Mutations of the NSD1 gene in familial cases of Sotos syndrome.

Case #	Nationality	Patients	Location	Mutation	Amino acids change	Reference
1	Israelite	Mother, son	Exon 5	2386–2389delGAAA	Frameshift	(8)
2	Finnish	Father, son	Exon 2	896delC	Frameshift	(9)
3	Turkish	Mother, daughter, son	Exon 23	6532delTGCCCCAGC	2178–2180delCPS	(10)
4	German	Father, two daughters	Exon 18	5737A>G	N1913D	(11)
5	German	Mother, son	Exon 2	607G>A	V203I	(11)
6	German	Mother, daughter, son	Exon 21	6241T>G	L2081V	(11)
			Exon 23	7576C>T	P2526S	
7	Finnish	Mother, two daughters	Exon 5	2333T>G	L778X	(12)
8	Israelite	Mother, daughter	Exon 6	3882delT	Frameshift	(12)
9	Israelite	Mother, son	Exon 10	4417C>T	R1473X	(12)
10	Israelite	Mother, son	Exon 13	4779–4781delTTTinsATTC	Frameshift	(12)
11	Israelite	Mother, daughter	Exon 13	4855T>C	C1619R	(12)
12	Israelite	Father, three sons	Exon 14	4987C>T	R1663C	(12)
13	Israelite	Mother, daughter	Exon 16	5375G>T	G1792V	(12)
14	German	Mother, son	Exon 22	6291delG	Frameshift	(12)
15	German	Father, son	Exon 22	6370T>C	C2124R	(12)
16	Turkish	Mother, son	Exon 23	6614A>G	H2205R	(12)
17	Dutch	Seven members of a three-generation family	Exon 23	6605G>A	C2202Y	(13)
18	Japanese	Mother, daughter, two sons	Intron 13	IVS13 + 1G>A	Skip exon 13	(14)
19	Spanish	Father, son	Exon 22	A6442delAGCGACCA	K2151fsX	(15)
20	German	Mother, son, daughter	Exon 23	6523T>A	C2175S	(16)
21	Irish	Eight members of a three-generation family	Exon 20	6115C>T	R2039C	(17)
22	Korean	Mother, daughter	Exon 22	6356delA	D2119V	(18)
23	Italian	Three members of a three-generation family	Exon 2	521T>A	V174A	Our case

*See Ref. (8–18)*.

Two out of our three patients (the mother and the maternal grandfather of the proband) showed the mutation of NSD1 gene associated with isolated overgrowth; this finding has never been reported previously among familial cases of SoS. Tatton-Brown et al. screened more than 300 parents of *NSD1*-positive patients identifying mutations in 11, all of whom showed clinical findings of SoS; in other words, they have not seen a case of incomplete penetrance for a confirmed pathogenic NSD1 gene mutation (12). On the basis of our data, we consider worthwhile to perform molecular analyses for SoS in both parents of *NSD1*-positive individuals even when showing isolated overgrowth (2).

According to Table 2, most of familial cases of SoS are caused by a point mutation of NSD1 gene; we also suggest a link between the location of the mutation in NSD1 gene and the reproductive fitness. Nearly half of familial cases of SoS presented with a microdeletion or a point mutation between exon 20 and exon 23: on the basis of this finding, we hypothesize that mutations between exon 20 and exon 23 are not likely to affect reproductive fitness. On the other side, more rare mutations in other coding exons, such as exon 2, are possibly associated with a defect of fertility and early recurrent miscarriages, as described in the mother of our proband.

Two Italian siblings with SoS phenotype (psychomotor delay, macrocrania, coarse face, and accelerated growth), possibly inherited from their father, had already been described previously but genetic tests for SoS were not available at that time, therefore our family represents the first Italian familial case of SoS with genetic confirmation (19). In addition to this, ours is the third report in which a missense mutation of NSD1 gene is found in three generations of the same family: van Haelst et al. described earlier seven members of a three-generation Dutch family with the mutation 6605G>A/C2202Y in exon 23 of NSD1 gene (13). Furthermore, Donnelly et al. described eight members of a three-generation Northern Irish family with SoS caused by the mutation 6115C>T/R2039C in exon 20 of NSD1 gene (17). Our report confirms that variation in phenotype is possible even for patients with the same abnormality of NSD1 gene, as previously reported (1, 12, 14, 17, 18). Thus, it is possible that stochastic factors, epigenetic mechanisms, intrauterine environment, functional polymorphisms in genes interacting with *NSD1*, and intrinsic variability in the regulation of downstream targets of *NSD1* may be responsible of a different expression profile of NSD1 gene; the resulting tissue-specific gene expression profile may possibly influence phenotypes of SoS (12). According to the previous hypothesis, genetic tests are not useful to predict the phenotype.

In conclusion, we identified an Italian family with a novel missense mutation in exon 2 of NSD1 gene; each member carried the same molecular abnormality with variable phenotypes and severity, from isolated overgrowth to classical SoS. The present family case report will contribute to the understanding of the correlation between genotype and phenotype in familial SoS.

## Ethics Statement

Written informed consent for the publication of the present case report was obtained from both parents of the proband and his maternal grandfather.

## Author Contributions

GL, FM, AG, FL, SL, and PG met the patient to each visit and wrote the present case report; AM performed the molecular analysis of NSD1 gene.

## Conflict of Interest Statement

The authors declare that the research was conducted in the absence of any commercial or financial relationships that could be construed as a potential conflict of interest.
